# Importance of the electrophoresis and pulse energy for siRNA-mediated gene silencing by electroporation in differentiated primary human myotubes

**DOI:** 10.1186/s12938-024-01239-7

**Published:** 2024-05-16

**Authors:** Mojca Pavlin, Nives Škorja Milić, Maša Kandušer, Sergej Pirkmajer

**Affiliations:** 1 Institute of Biophysics, Faculty of Medicine, University of Ljubljana, Vrazov Trg 2, 1000 Ljubljana, Slovenia; 2 Group for Nano and Biotechnological Applications, Faculty of Electrical Engineering, University of Ljubljana, Ljubljana, Slovenia; 3 Institute of Pathophysiology, Faculty of Medicine, University of Ljubljana, Zaloška 4, 1000 Ljubljana, Slovenia; 4 Institute of Anatomy, Faculty of Medicine, University of Ljubljana, Korytkova 2, Ljubljana, Slovenia; 5 Pharmacy Institute, Faculty of Pharmacy, University of Ljubljana, Ljubljana, Slovenia

**Keywords:** Primary human myotubes, siRNA, Electrotransfection, Electrophoresis, Mechanisms, Gene silencing, Electroporation

## Abstract

**Background:**

Electrotransfection is based on application of high-voltage pulses that transiently increase membrane permeability, which enables delivery of DNA and RNA in vitro and in vivo. Its advantage in applications such as gene therapy and vaccination is that it does not use viral vectors. Skeletal muscles are among the most commonly used target tissues. While siRNA delivery into undifferentiated myoblasts is very efficient, electrotransfection of siRNA into differentiated myotubes presents a challenge. Our aim was to develop efficient protocol for electroporation-based siRNA delivery in cultured primary human myotubes and to identify crucial mechanisms and parameters that would enable faster optimization of electrotransfection in various cell lines.

**Results:**

We established optimal electroporation parameters for efficient siRNA delivery in cultured myotubes and achieved efficient knock-down of HIF-1α while preserving cells viability. The results show that electropermeabilization is a crucial step for siRNA electrotransfection in myotubes. Decrease in viability was observed for higher electric energy of the pulses, conversely lower pulse energy enabled higher electrotransfection silencing yield. Experimental data together with the theoretical analysis demonstrate that siRNA electrotransfer is a complex process where electropermeabilization, electrophoresis, siRNA translocation, and viability are all functions of pulsing parameters. However, despite this complexity, we demonstrated that pulse parameters for efficient delivery of small molecule such as PI, can be used as a starting point for optimization of electroporation parameters for siRNA delivery into cells in vitro if viability is preserved.

**Conclusions:**

The optimized experimental protocol provides the basis for application of electrotransfer for silencing of various target genes in cultured human myotubes and more broadly for electrotransfection of various primary cell and cell lines. Together with the theoretical analysis our data offer new insights into mechanisms that underlie electroporation-based delivery of short RNA molecules, which can aid to faster optimisation of the pulse parameters in vitro and in vivo.

**Supplementary Information:**

The online version contains supplementary material available at 10.1186/s12938-024-01239-7.

## Introduction

Gene transfer using electroporation, is an efficient non-viral approach for gene therapy [1, 2]. Locally delivered electric pulses transiently increase membrane permeability (electroporation) which enables transfer of small molecules and nucleic acids into cells [2–7]; however, the complete description of the process is still missing [5, 6, 8–17]. Electrotransfection has been extensively used for delivery of plasmid DNA (pDNA) and short RNAs in various experimental biomedical applications such as gene therapy and DNA vaccination including vaccine development against COVID-19 [18, 19], treatment of cancer [20–29] and infectious diseases [30, 31]. Compared to viral transduction it has lower efficiency yet its important advantage is that it has minimal side effects compared to viral transduction [32, 33] and can boost even superior response [34].

Skeletal muscles, which represent large part of body mass are particularly attractive as a target tissue for electrotransfer due to high accessibility for the delivery of electric pulses and significant capacity for protein synthesis and regeneration [20, 21, 35–38]. Electrotransfection of skeletal muscle is explored for treatment of muscle disorders [39] and widely used for systemic delivery of therapeutic proteins [20, 25, 26, 26, 40–43] [44–46], and DNA vaccination [27, 28, 34].

Primary human skeletal muscle cells are a convenient and widely used model for the investigation of various aspects of skeletal muscle function [47, 48]. This experimental approach is particularly potent in combination with small interfering RNA (siRNA)-mediated gene silencing, which allows functional assessment of target proteins under-well controlled conditions in cell culture [23, 49–53]. Depending on the study, experiments are carried out either on proliferating myoblasts [52, 54] or on the postmitotic fused myotubes [47, 48, 53, 55]. However, while gene silencing in myoblasts is highly efficient, myotubes are more resistant to transfection with lipid-based transfection agents as well as calcium phosphate [56], which may limit the extent to which the abundance of the target protein is suppressed. To overcome these challenges, we aimed to establish an electroporation-based protocol for siRNA-mediated gene silencing in cultured human myotubes.

Electroporation is a threshold phenomenon and occurs only above the critical transmembrane potential (*U*_*c*_) [2, 6–8], which is in the range of 0.2–1 V for most cells, but it is unknown for cultured human myotubes. When the electric field is applied to a living cell, a transmembrane voltage (*U*_*m*_) is induced across the plasma membrane. For a spherical cell with a nonconductive membrane, *U*_*m*_ can be calculated with the simplified Schwan equation (see Appendix).

1 $$U_{m} = {\text{1}}.{\text{5}}\,E\,R\,cos\,\vartheta.$$

Once *U*_*m*_ exceeds *U*_*c*_ electropermeabilization in the plasma membrane occurs and enables diffusion of molecules into the cell (Additional file 1: Fig. S1) [6, 9, 16] through the permeabilized surface that is directly determined by the electric field and *U*_*c*_ (Additional file 1: Eqs. S4 and S12)*. U*_*m*_ is linearly dependent on the local electric field *E* and on the size and shape of the cell. Myotubes are elongated cells [48, 57] which can be addressed mathematically using the analytical solution for spheroidal cells (see Appendix) [58]. The maximal* U*_*m*_ is reached when the myotube is oriented parallel to *E*, in that case *U*_*m*_ is proportional to the size of a cell in the *z* direction (*R*_*1*_): *U*_*max*_ = *R*_*1*_* E* (Eq. S10). The electropermeablization threshold *U*_*c*_ is, therefore, reached first in myotubes oriented in parallel to the applied electric field and the critical electric field is when permabilization is reached:

2 $$E_c = \frac{U_c }{{R_1 }}.$$

As* U*_*c*_ depends on pulse duration (*t*_*E*_), number of pulses and repetition frequency, so the efficiency of siRNA or DNA electrotransfer in cultured human myotubes is likely a complex function of the *E* and pulse parameters [3, 5, 11, 59–61], where in addition to membrane electropermeablization, also electrophoretic driving force acting on DNA and RNA molecules was shown to be important [3, 37, 59, 62, 63]. However, a protocol that enables effective delivery of siRNA, may reduce the viability of target cells, thus reducing the overall efficiency of the experimental approach.

We experimentally and theoretically evaluated the permeabilization, siRNA-mediated gene silencing, and viability in cultured human myotubes as a function of the main electroporation parameters. Collectively, our results show that electroporation effectively produces gene silencing in cultured human myotubes and provide new theoretical insights regarding the mechanisms that underlie electrotransfer of siRNA.

## Results

### Electropermeabilization of cultured human myotubes

Electropermeabilization of the plasma membrane is a precondition for delivery of siRNA. To determine the optimal conditions for siRNA delivery into cultured human myotubes, electropermeabilization and viability experiments were performed with trains of 8 × 2 ms and 8 × 5 ms square pulses (1 Hz repetition frequency) and various electric field strengths (*E*) using RPMI-1640 medium as the electroporation buffer. These pulsing protocols (8 × 2 ms and 8 × 5 ms) were selected on the basis of our preliminary study in myoblasts and myotubes, where we tested a variety of pulsing conditions, from very long pulse durations (8 × 10 ms) to shorter (4 × 200 µs) pulses; however, at both extremes, the electrotransfection was very low for the tested voltages [64, 65]. In these pilot experiments we have also tested combinations of high-voltage and low-voltage pulses, but similarly transfection and viability were relatively low. The electroporation buffer was also chosen based on our experience with cultured human myoblasts [64, 65]. In the present study in myotubes we first tested various pulses (also 8 × 1 ms and 8 × 10 ms) pulses in the range of 0.05 to 0.6 kV/cm and bipolar pulses 8 × 2 ms in the range of 0.1 to 0.6 kV/cm. Optimal viability and permeabilization were obtained for 8 × 2 ms and 8 × 5 ms pulses, so these pulsing protocols were used in the present study.

**Fig. 1:**
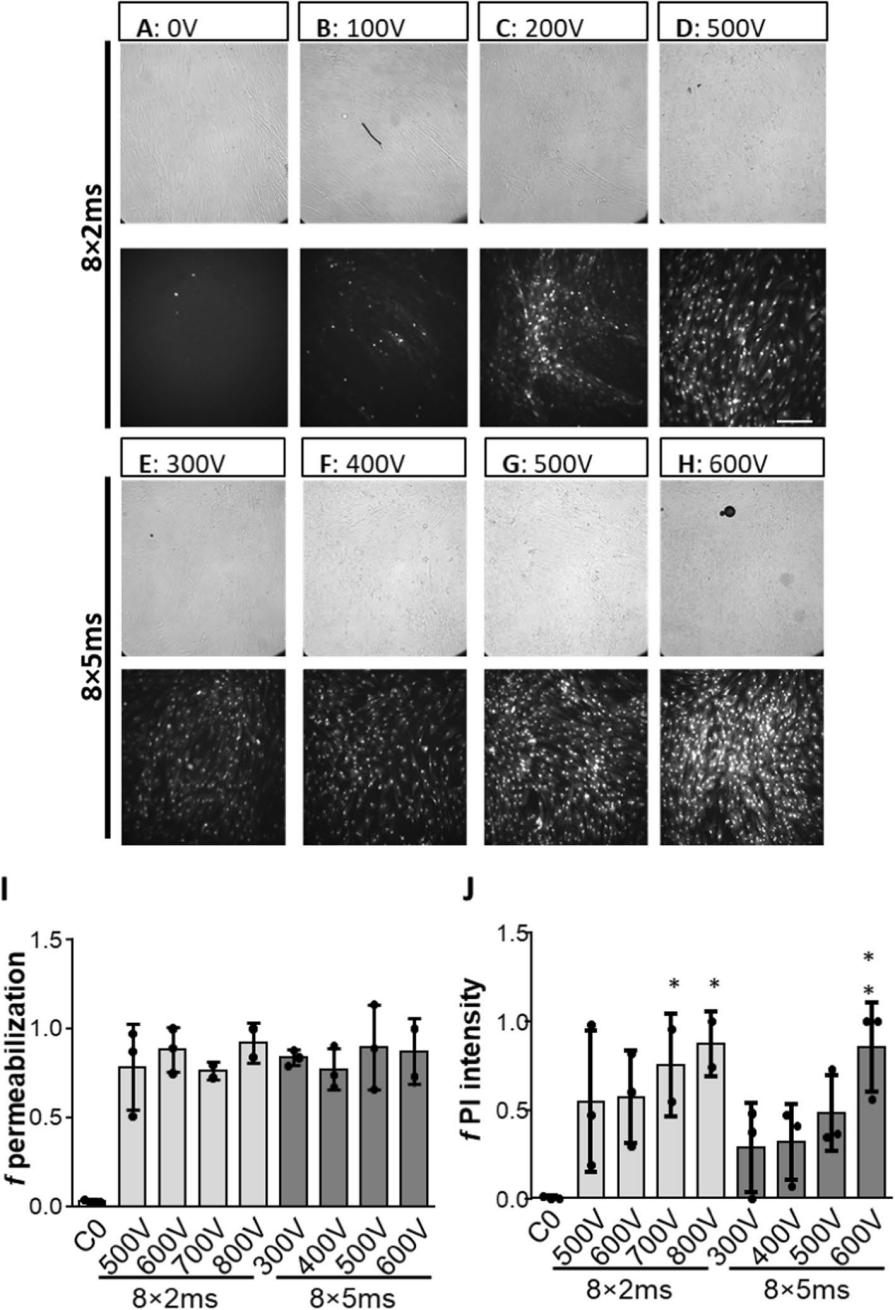
Visualization and quantification of electropermeabilization of cultured human myotubes. Myotubes were electroporated in the presence of 0.15 mM fluorescent dye PI using a train of 8 × 2 ms (**A**–**D**) or train of 8 × 5 ms pulses (**E**, **H**) with increasing voltages. Bright field (upper) and fluorescent images (lower) were taken 5–10 min after electroporation (**A**–**H**), the scale bar is 200 µm (**H**). The cells in the control sample (C0) were exposed to PI but no pulses were applied (*U* = 0 V). The fraction of permeabilized cells (*f* permeabilization) was determined from the number of PI positive cells nuclei divided to the number of all counted cells nuclei (**I**). The extent of electropermeabilization was determined by analysing the fluorescence intensity of PI of the microscopic images *I*_PI_ [a.u.]. Intensity of PI fluorescence (*f PI* intensity-see Eq. 8) normalized to maximum PI fluorescence intensity is shown **J**. Results are presented as mean ± SD of three independent experiments (*N* = 3). *P ≤ 0.05 vs. C0

The lowest *E* which caused permeabilization (*E*_*c*_) was below 0.105 kV/cm (*U*_c_ < 100 V) as determined using small molecule PI [5], and was lower with the 8 × 5 ms than the 8 × 2 ms protocol. While only a few myotubes were permeabilized when field strengths near the *E*_*c*_ were applied, increased field strengths above the threshold increased the percentage of permeabilized cells (*f permeabilization*) and PI fluorescence intensity (Fig. 1A–H). The majority of myotubes (> 75%) was permeabilized with voltages 500 V and above with the 8 × 2 ms pulsing protocol (Fig. 1D, I). With the 8 × 5 ms protocol, permeabilization was 75–95% with voltages 300 V or higher (Fig. 1I).

The extent of myotube electropermeabilization was also estimated by normalized PI fluorescence intensity (*f* PI *Intensity*, Fig. 1J), which increased in parallel with applied voltages (electric field strength). Results were similar for both pulsing protocols, but the trend of increasing PI intensity was more pronounced for the 8 × 5 ms pulses. Taken together, our results indicate that PI fluorescence intensity increased with increasing voltage (Fig. 1J) without an attendant increase in the fraction of permeabilized cells (Fig. 1I), indirectly suggesting that higher voltage increased permeabilized surface area (*S*_*c*_) and consequently diffusion of PI.

### Silencing of HIF-1α in human myotubes with siRNA electroporation

**Fig. 2:**
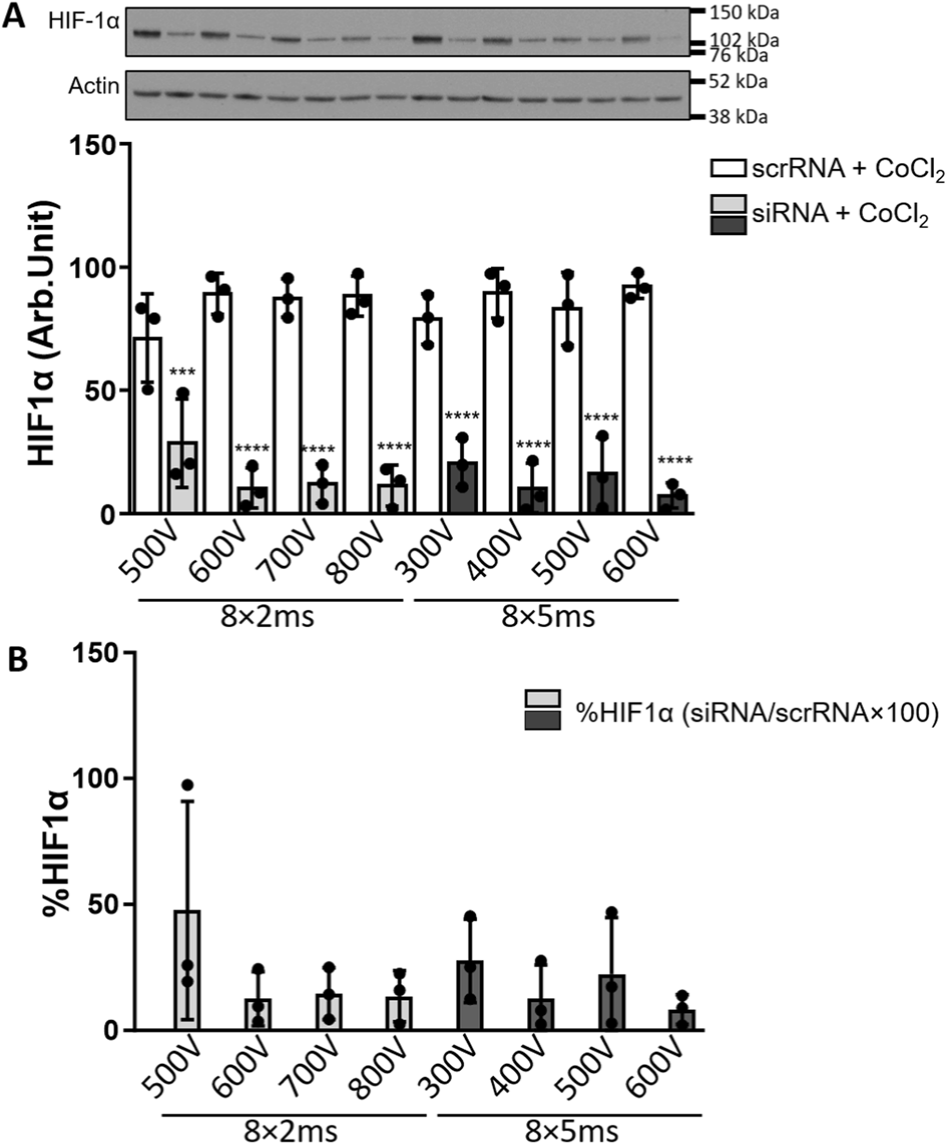
Silencing of HIF-1α protein in human myotubes after siRNA delivery using electroporation determined with Western Blot. HIF-1α siRNA electroporation significantly reduced the quantity of HIF-1α protein. **A** HIF-1α bands of a representative experiment are shown on top, actin bands are shown as a loading control. HIF-1α protein expression [AU] is shown in A. In **B** percentage of HIF-1α expression is shown normalized to the scr control-electroporated sample with scrRNA; %HIF-1α = HIF-1α siRNA/HIF-1α scrRNA. Results are presented as mean ± SD of three independent experiments (*N* = 3). The statistical significance is shown as analysed by ANOVA with Bonferroni post-hoc correction, we compare the abundance of HIF-1α after electrotransfer with scrRNA vs siRNA for each pulsing parameter ***P ≤ 0.001; **** *P* ≤ 0.0001

To optimize electroporation pulsing protocols for siRNA-mediated gene silencing, we selected hypoxia inducible factor-1α (HIF-1α) as our target. HIF-1α is a major transcriptional regulator of oxygen homeostasis [66] that is markedly upregulated in hypoxia and stimulates adaptive transcriptional responses in human cells [52, 64, 67]. To silence HIF-1α, we used siRNA, which was previously shown to cause significant knock-down of HIF-1α in cultured human myoblasts using lipofection and/or electrotransfection [52, 64, 68]. Myotubes were treated with CoCl_2_ (250 μM for 4 h) 24 h after electrotransfection with siRNA against HIF-1α (siHIF-1α) or scrambled siRNA (siSCR) (Fig. 2). In CoCl_2_-treated myotubes that had been electrotransfected with siHIF-1α, the abundance of HIF-1α was reduced by ~ 80% on average (Fig. 2A, B). While silencing appeared to be least efficient with 8 × 2 ms pulses of 500 V (HIF-1α reduced by $\sim$53%, siHIF-1α vs. siSCR, *P* < 0.05) and most efficient with 8 × 2 ms pulses of 600V (HIF-1α reduced by $\sim$92%, siHIF-1α vs. siSCR, *P* < 0.05), differences between different voltages did not reach the level of statistical significance.

### Viability of cultured human myotubes after electroporation

**Fig. 3:**
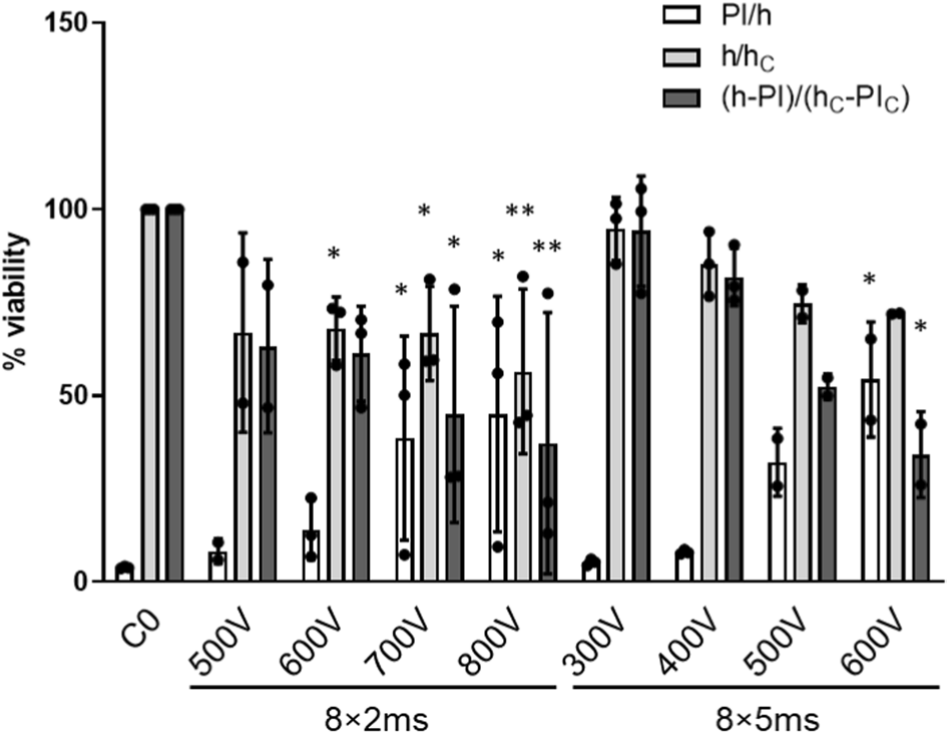
The effect of electroporation parameters on viability of primary human myotubes. The percentage of viability was obtained as the number of viable myotube nuclei in the treated sample (*h–PI*) and the number of viable myotube nuclei in the negative control (*h*_*C*_ *-PI*_*C*_): %viability = (*h–PI*)/(*h*_*C*_*–PI*_*C*_) × 100 (Eq. 9). The percentage of PI positive (*PI/h*) and of all Hoechst positive myotube nuclei normalized to control sample are also presented (*h*/*h*_*C*_). Myotubes were electroporated using 8 × 2 ms and 8 × 5 ms with different applied voltage. Cells in the control sample (*C0*) were not electroporated (*U* = 0 V). Results are presented as mean ± SD of three independent experiments (*N* = 3)

Viability was assessed 24 h after exposure (Fig. 3) using the same pulsing protocols as for electrotransfection. The relative number of PI-positive nuclei (dead nuclei) increased with the applied voltage and pulse duration up to 50% for the highest voltages, in parallel the total number of Hoechst-positive nuclei decreased. The percentage of viability was determined as the ratio between the number of viable myotube nuclei in the treated sample and the number of viable myotube nuclei in the negative control %viability = (*h-PI*)/(*h*_*C*_*-PI*_*C*_) × 100 (see M&M). Viability decreased with increasing voltage and pulse duration, dropping to 35% for 8 × 2 ms pulses at *U* = 800 V, and to 30% for the 8 × 5 ms at *U* = 600 V. The highest viability (95%) was detected at the lowest voltage (300 V) of the 8 × 5 ms protocol (Fig. 3).

### Silencing yield depends on the electroporation protocol

**Fig. 4:**
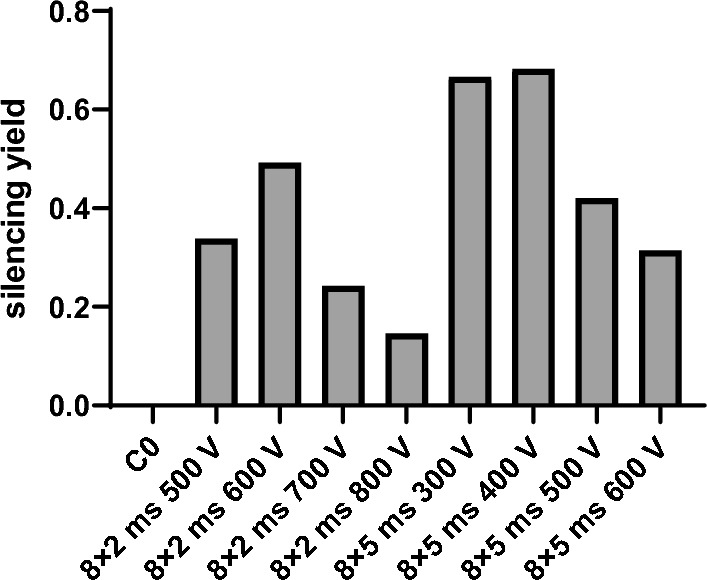
The effect of electroporation parameters on the silencing yield of primary human myotubes. The silencing yield was obtained from the viability and efficiency of silencing: *f silencing yield* = *f viability* × *f silencing*. The calculated averages are shown

The yield of successfully silenced myotubes depends on viability and silencing efficiency:

3 $$f \, silencing \, yield \, = \, f \, viability \, \times \, f \, silencing$$

where *f viability* is the measure of viable myotubes (*f viability* = % viability/100) and *f silencing* is the efficiency of silencing knockdown, calculated as: *f silencing* = 1-siHIF1α/siSCR, where siHIF-1α and siSCR indicate the abundance of HIF-1α protein after transfection with siRNA against HIF-1α after transfection with non-targeting scrambled siRNA (control sample of silencing). The highest silencing yield (around 70%) was obtained with 8 × 5 ms 300 V and 8 × 5 ms 400 V pulses (Fig. 4), where both very high silencing and high viability (> 80%) was achieved. For other parameters, the viability dropped significantly (below 40%), resulting in a decrease in the silencing yield.

### Diffusion, electrophoresis and energy of the electric pulses - a theoretical analysis

#### Diffusion of siRNA

The mobility of charged molecules such as RNA are described by Nernst–Planck equation, which incorporates both diffusion and electrophoresis [5, 69–71]. Before pulse application diffusion dominates, while during pulse application strong electric field results in an electrophoretic force that drags siRNA in the opposite direction of the applied electric field. In in vitro experiments, such as in our case we can assume that before electroporation siRNA is homogeneously distributed around the cells. In case of in vivo electrotransfection siRNA slowly diffuses from the injection site, with the diffusion constant being *D* ~ 10^–6^ cm^2^/s [70, 72]. During the electric pulses, the displacement due to diffusion can be neglected and we can, therefore, estimate the displacement only due to electrophoresis [64, 73].

#### Electrophoresis

The electrophoretic drag is important since it enables the accumulation of DNA or siRNA molecules at the cell membrane as well it enables potential transfer across formed electropores. The electrophoretic force affects the efficiency of electrotransfer [3, 5, 62–64, 70, 74, 75] by dragging the negatively charged RNA or DNA molecules toward the cathodic side of the plasma membrane. Additionally, electrophoresis effectively provides the energy to overcome the repulsive forces between the negatively charged plasma membrane and nucleic acids. Consequently, the electric field during the pulses not only induces permeabilization of the membrane but also increases the number of DNA/RNA molecules interacting with the plasma membrane and directly provides additionally force acting on the charge molecules thus promoting their delivery into cells [5, 65]. The electrophoretic force (*F*_*e*_) is proportional to the applied electric field *E* and the effective charge of the given molecule (*e*_eff_):

4 $$F_E = e_{{\text{eff}}} E ,$$

where *e*_eff_ is proportional to the length of a DNA or RNA molecule [76]. In the cell culture media or other electroporation buffer the effective charge *e*_eff_ is much smaller compared to *e* in water due to screening effects of the counterions.

**Fig. 5:**
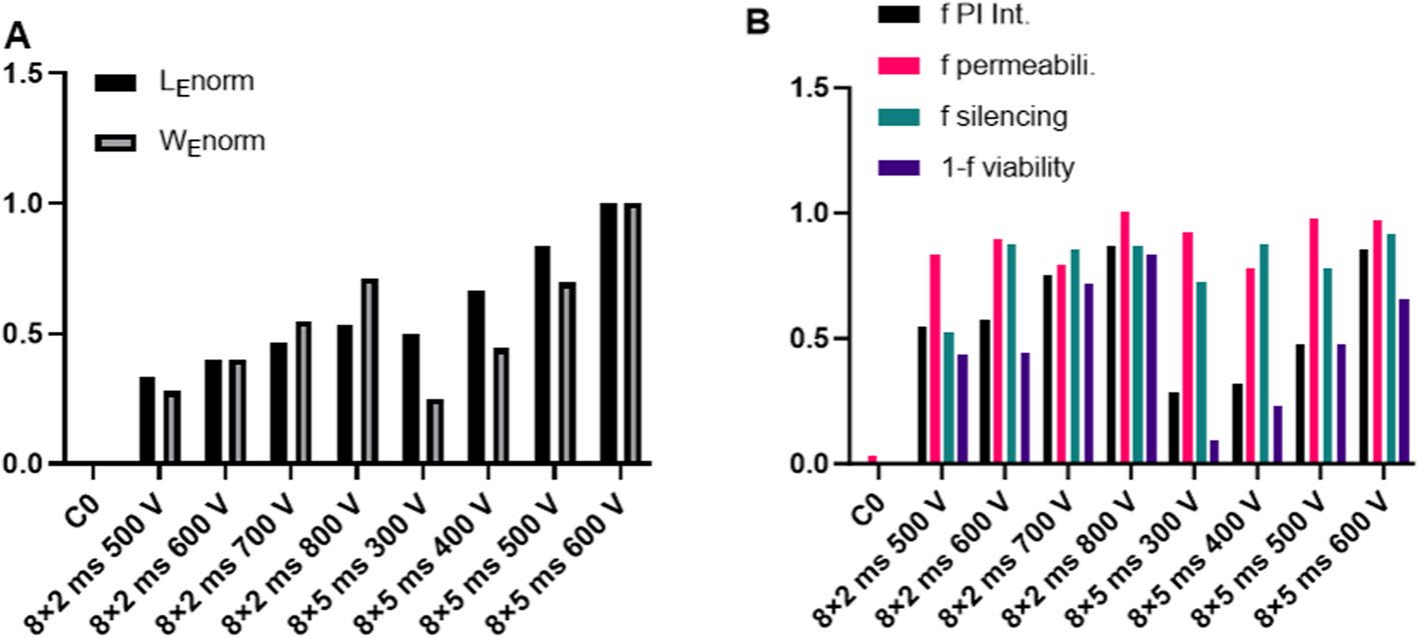
The comparison of the theoretically calculated electrophoresis and electric energy of the pulses, with the experimentally determined permeabilization, silencing efficiency and viability. The theoretically calculated electrophoretic displacement *L*_*E*_* norm* = *L*_*E*_* /L*_*E*_* max* and normalized electric energy of the pulses *W*_*E*_ = *W*_*E*_*/W*_*E*_* max* (**A**). Comparison of the fraction of permeabilized cells (*f permeabil.*)*,* extent of permeabilization measured by PI fluorescence intensity (*f PI Intensity*), silencing efficiency (*f silencing*) and decrease in viability (1-*f viability*) (**B**). For the experimental data, values of the means are presented

In the following, we will present calculations of electrophoresis for siRNA molecules. For a double stranded siRNA in water the charge is: *e* =*2e*_*0*_ per base pair × *N*_bp_, in our specific case* N*_bp_ = 21. During the pulse application the electrophoretic force *F*_*e*_ drags siRNA in the opposite direction of the field. The electrophoretic displacement (*L*_E_) during pulse application can be calculated [5, 64, 69, 73]:

5 $$L_{\text{E}} = v\;t_{\text{E}} \; = \mu \,E\,t_{\text{E}},$$

where *v* is the velocity of a siRNA molecule that is in steady state proportional to mobility *µ* and *t*_E_ is total pulse duration of *N* pulses is:* t*_E_ = *t*_1_ × *N*, where we assume that during each pulse approximately the same electrophoretic displacement occurs. The mobility of siRNA molecule *µ* can be calculated from:

6 $$\mu = \frac{{e_{{\text{eff}}} }}{{6\pi \eta R_{\text{g}} }},$$

where *η* is viscosity of media and *R*_g_ is the radius of gyration of the siRNA molecule. RNA molecules are polyelectrolytes that behave as polymer chains for which the radius of gyration can be estimated; for a 21 bp long dsRNA *R*_g_ is approximately 1.8 nm [76]. The effective charge *e*_eff_ in a specific physiological media is usually not known, therefore, mobility has to be alternatively estimated from measurements of RNA mobility in similar conditions. We used data from literature [72] of measured mobility of RNA molecules in similar media and by taking into account dependence on the number of base pairs we obtain: *µ* ≅ 0.5 × 10^–4^ cm^2^ V^−1^ s^−1^. Using Eq. 6, we estimated the electrophoretic displacement *L*_E_ for different field strengths and the total pulse duration for our specific pulsing protocols. The results are presented in Fig. 5A normalized to the maximal value of *L*_E_ for the selected pulses.

#### The energy of the electric pulses

We further calculated the energy of the electric pulses *W*_E_ (Fig. 5A), which can be also one of determinant factors for efficient electrotransfection [61] and viability (Fig. 3). Among other factors the Joule heating is directly proportional to the electric energy of the pulses. The normalized electric energy was calculated as *W*_*E*_*norm* = *W*_*E*_* / W*_*E*_*max* = *t*_*E*_ × *E*^*2*^* /* (*t*_*E*_ × *E*^*2*^)_*max*_. Since *W*_*E*_ is proportional to the square of the applied electric field *E* (and square of *U),* the electric energy increases more steeply with the voltage than the electrophoretic displacement that depends linearly on both parameters (*t*_*E*_ and *E*).

Next, we ask ourselves a question how these theoretical parameters relate with the silencing efficiency and silencing yield. In Fig. 5, we compare the theoretically calculated electrophoretic displacement and normalized electric energy (Fig. 5A) with the experimentally determined permeabilization, silencing efficiency (Fig. 5B), and silencing yield (Fig. 4). Similar values of silencing efficiency for different pulsing protocols showed similar behaviour as the fraction of permeabilization for which also the values were almost constant (between 80 and 100%). It can be also observed, that the silencing efficiency has a different functional dependency as *W*_*E*_ or electrophoretic displacement (*L*_E_).

We performed our analysis on an in vitro system, but the main conclusions are valid also for tissues. Since siRNA is relatively small molecule and its radius gyration (*R*_g_ ~ 1.8 nm) is smaller than pores sizes in the extracellular matrix in tissue (20–200 nm), the electrophoresis and diffusion of siRNA will be hindered much less compared to larger plasmid DNA molecules (Zimm model) [69, 70, 72, 77]. Therefore, we expect that in tissues the electroporation parameters that enable delivery of small molecules will enable also efficient delivery of siRNA, however, the silencing efficiency could be affected due to poor RNA stability. The above analysis is valid also for other short RNA molecules such as miRNA.

### Electrotransfection of plasmid DNA

**Fig. 6:**
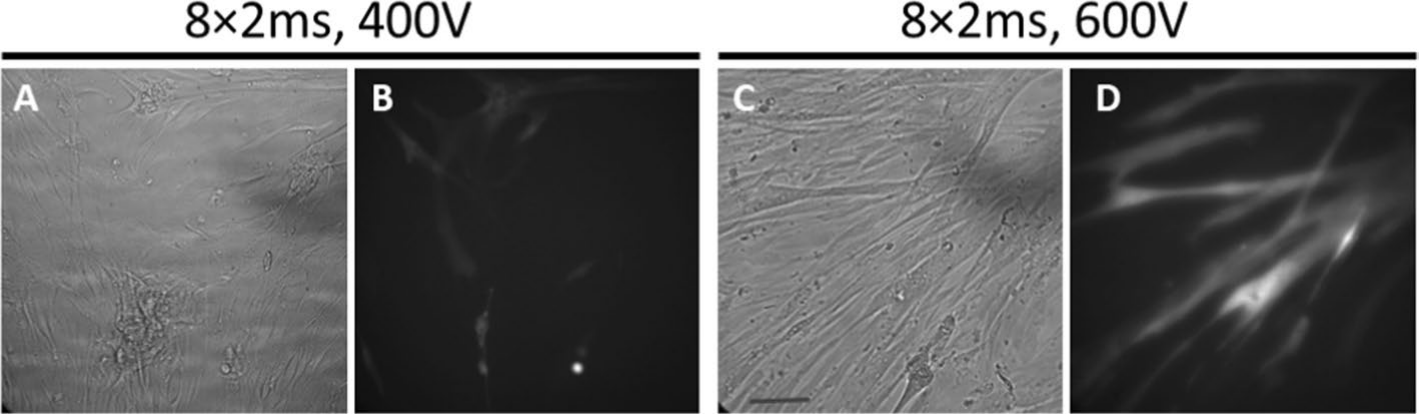
Electrotransfection of primary human myotubes. Cells were electroporated in the presence of 40 µg/ml pEGFP using a train of 8 × 2 ms pulses with 400 V (**A**, **B**) and 600 V (**C**, **D**). Bright field (**A**, **C**) and fluorescent images (**B**, **D**) of GFP expression were recorded 24 h after electroporation. The percentage of GFP-positive myotubes for 8 × 2 ms was 5.8 ± 1.55% at 400 V (**B**) and 11.5 ± 0.8% at 600 V (**D**), the values are mean ± SEM. The scale bar is 40 µm

Next, we examined whether we can use the optimized pulsing protocols for delivery of siRNA also for delivery of plasmid DNA, which are much larger molecules. Based on the presented results and our previous experience of pDNA and siRNA electrotransfer into human myoblasts [64, 65], we used 8 × 2 ms pulses at *U* = 400 V and 600 V to deliver pDNA encoding green fluorescent protein (pEGFP-N1) (Fig. 6). We have obtained relatively low percentage of GFP expressing myotubes with 8 × 2 ms pulses at *U* = 400V (5% GFP positive cells) after 24 h (Fig. 6A, B). In contrast, a significant percentage (11%) of myotubes expressed GFP after 24 h (Fig. 6C, D) for higher applied voltage *U* = 600 V for the same train of pulses (8 × 2 ms).

## Discussion

Gene silencing in cultured human myotubes is an attractive option to investigate physiologically and pharmacologically relevant targets [78, 79], including HIF-1α [52, 64, 68]. Here, we developed a new electroporation-based protocol for efficient siRNA-mediated gene silencing in cultured human myotubes. We further theoretically analyze the crucial mechanism to identify crucial parameters that could help to design future efficient electroporation protocols for siRNA delivery in various cell types.

Some myotubes were permeabilized already at 100 V (Fig. 1), meaning that *E*_*c*_, a prerequisite step for siRNA delivery [64, 74], was relatively low (below 0.1 and 0.05 kV/cm). This is consistent with the specific geometry and size of myotubes, which are thin and elongated [48, 57]. The induced transmembrane potential (*U*_*m*_), which determines the extent of electropermeabilization, increases linearly with *E* and the size of the cell. Myotubes, which are oriented along the applied electric field should, therefore, should be permeabilized already at low threshold electric field *E*_*c*_ (see Eq. 2 and Appendix for details): *E*_c_ = *U*_c_ / *R*_1_.

We performed gene silencing of HIF-1α with two pulsing protocols: (1) 8 × 2 ms with the applied electric fields *E* = [0.52, 0.63, 0.73 and 0.84] kV/cm and (2) 8 × 5 ms, *E* = [0.315, 0.42, 0.52 0.63] kV/cm with 1 Hz repletion frequency. Interestingly, *E*_*c*_ for silencing in myotubes was not very different from the *E*_*c*_ in myoblasts [64] despite a much larger *R*_*1*_, suggesting that other biological factors importantly determine *E*_*c*_ through different *U*_*c*_ in myotubes. Gene silencing of HIF-1α was particularly effective with 8 × 5 ms pulses at *U* = 600 V, which reduced the abundance of HIF-1α by 92% (Fig. 2). While HIF-1α silencing was only slightly less pronounced at lower voltages and/or shorter pulses (500 V 8 × 2 ms and 300 V 8 × 5 ms) (Fig. 2), higher voltages and longer pulses significantly decreased viability of myotubes (Fig. 3) after 24 h. We have to stress that at later time points viability could be further reduced, however, as we have previously shown [64], most of the decrease in viability occurs in first 24 h.

Electroporation induces stable pores in the plasma membrane [8, 16, 80–82], which not only enable siRNA delivery, but also cause leakage of ions and other molecules, thus disrupting cellular homeostasis. Thus, if *E* is too high for a given pulse duration, the uptake of siRNA into myotubes is high, but the yield of silenced myotubes is low because they are irreversibly damaged (Figs. 3, 5B). Depending on the application, the optimal balance between preserved viability and efficient delivery is crucial. It is, therefore, interesting to analyze with parameters relate with the silencing efficiency, silencing yield and viability. Viability and consequently the silencing yield (Fig. 5) were decreasing with the increase in the total energy of pulses (*W*_*E*_) (Fig. 5A) that can be explained primarily with the Joule heating (*Q*), which is directly proportional to the electric energy of pulses:

7 $$Q \propto \sigma \times t_E \times\ E^2$$

where *t*_*E*_ is the total duration of the electric pulses and *σ* is conductivity of the electroporation media [8, 16]*.* In a highly conductive medium, Joule heating would be the main limiting factor for longer and higher electric pulses, meaning that the medium should be selected carefully. The conductivity of our RPMI-1640 medium is 1.34 S/m.

Permeabilization of cultured human myotubes was assessed using PI, raising the question of whether such a small molecule could be used to predict siRNA electrotransfer efficiency. Regardless of the pulsing protocol the fraction of PI-positive nuclei (Fig. 1I) was above 0.75, while the intensity of PI fluorescence (Fig. 1J) tended to depend on the applied voltage, especially for the 8 × 5 ms protocol. Theoretical analysis of electropermeabilization (see Additional file 1: Appendix) shows that larger surface area (*S*_*c*_) is permeabilized with the increasing voltage (Eq. S12) [58], which is paralleled by increased number and size of pores within the *S*_*c*_ [5, 7, 8, 82, 83]. Accordingly, *S*_*c*_ and the total surface area of electropores increases when *E*_*c*_ is exceeded, which in turn allows faster diffusion of molecules into cells, consistent with the increase of PI fluorescence intensity (Fig. 1J).

Interestingly, silencing efficiency with different pulsing protocols was relatively comparable (Fig. 5B), correlating with the fraction of permeabilization (Fig. 1I) rather than with the extent of permeabilization, as assessed as PI fluorescence intensity (Fig. 1J). The radius of gyration of 21 bp siRNA is around 1.8 nm and is, therefore, smaller than electropores, whose radii range from 2 nm to tens of nm [82] and we could, therefore, expect them to diffuse freely into myotubes. Nevertheless, the extent of electropermeabilization did not follow the dependence of the silencing efficiency obtained with the specific pulse parameters. Namely, for 8 × 5 ms pulses PI intensity increased with the applied voltage, while silencing efficiency increased only moderately in agreement with the study demonstrating that siRNA is not entering the cells just by pure diffusion [64, 74]. Most likely the extent of silencing does not linearly depend on the number of siRNA molecules that enter the myotubes, but also depends on the process of siRNA transport inside the cell. This suggests that the efficiency of the silencing itself may not be improved by increasing the intracellular siRNA concentration once the threshold concentration for silencing is reached.

High permeabilization (> 75%) and high HIF-1α silencing efficiency for the indirectly suggest that electropermeabilization is a precondition for the siRNA delivery into myotubes, as observed in other types of cells [64, 74] and also for delivery of pDNA [5, 61, 83]. However, the mechanisms of the siRNA delivery likely differ from those of pDNA, which are much larger molecules. For instance, while the formation of DNA-membrane complex was observed during electrotransfer, no such complex was observed in the case of siRNA [74]. Despite the differences, we hypothesized that optimization of the siRNA delivery into myotubes should provide a basic electroporation protocol that could enable faster optimization for pDNA electrotransfer. Consistent with this idea, pEGFP was successfully introduced into primary human myotubes using 8 × 2 ms at *U* = 600 V (Fig. 6). Thus, our results suggest that pulsing protocol for pDNA delivery is likely close to the optimal parameters for the siRNA delivery and vice versa.

Analysis of PI fluorescence intensity, efficiency of silencing, the energy of the pulses (*W*_*E*_) and electrophoretic displacement (*L*_*E*_) shows that the theoretical parameters (*W*_*E*_*, L*_*E*_) or permeabilization cannot directly predict the efficiency of silencing on their own. *W*_*E*_ strongly depends on the applied electric field (*W*_*e*_ ∝ *E*^*2*^), while silencing efficiency does not follow such dependence. However, electric energy is crucial to estimate the effects on decreased viability (1-*f viability*), which it follows very closely (Fig. 5B). Consequently *W*_*e*_ also directly affects silencing yield (Fig. 4). *L*_*E*_ (Fig. 5A) cannot solely describe the silencing efficiency as it will increase even when almost complete silencing is achieved. The electrophoretic displacement of siRNA molecules *L*_*E*_ is directly proportional to the applied *E* and *t*_*E*_ that govern electrophoretic displacement of negatively charged siRNA toward the plasma membrane and potentially through the hydrophilic electropores. Since the membrane thickness is 5–7 nm, which is in a range of the *L*_*E*_ for few ms pulses, electrophoretic drag could enable the transfer of siRNA molecules across the plasma membrane. This transfer could be described similarly to electric field-driven translocations of polynucleotides through synthetic pores used for nucleotide sequencing [3, 84]. However, up to now no direct visualization of electrotransfer of siRNA or pDNA on a molecular level is available to prove this explanation.

In general, silencing efficiency is a complex function of many physical and biological parameters. This is not surprising since electrotransfer of siRNA and other short RNAs is a complex and dynamic process where electropermeabilization, electrophoresis, RNA translocation, and viability are all dependent on pulsing parameters. However, despite this complexity, we showed that the threshold for electropermeabilization estimated using small molecule, such as PI, can be used as a starting point for optimization of electroporation parameters for delivery of short RNA molecules in myotubes, especially if viability is assessed in parallel.

## Conclusions

Here, we show that electroporation-based siRNA delivery is an efficient method for gene silencing in cultured human myotubes. Myotubes are susceptible to stress imposed by electroporation procedure; however, with optimization we achieved efficient knock-down of HIF-1α while preserving myotube viability. The optimized experimental protocol provides the basis for application of electrotransfer for silencing of various target genes or for delivery of miRNA molecules in cultured human myotubes, which will enable investigation of different aspects of skeletal muscle (patho)physiology and pharmacology. We show that permeabilization is a crucial step for siRNA electrotransfer in myotubes and that silencing yield with electrotransfer will be more optimal with pulses with lower electric energy. Finally, together with the theoretical analysis our experimental data offer new insights into mechanisms that are important for electrotransfer of short RNA molecules more generally, in primary human myotubes as well as in other cells in vitro, thus opening new opportunities for further optimization of electroporation-based gene silencing.

## Material and Methods

### Cultured human myotubes

Use of human skeletal muscle cells was evaluated by the Republic of Slovenia National Medical Ethics Committee (71/05/12 and 0120-698/2017/4). Donors were free of neuromuscular disease. We have tested electrotransfection and assessed viability after electroporation in four different donors: 19F, 33M, 45F, 70M (number indicates age, M/F gender). In gene silencing experiments donor 24F was used. Skeletal muscle cell cultures were prepared as described [57, 67, 85]. Briefly, primary culture was prepared from samples of the *semitendinosus* muscle from subjects with knee injury. Muscle sample (surgical waste) was obtained with written consent during routine orthopaedic operations of the knee. Muscle tissue was cleaned of connective and adipose tissue, cut to small pieces, and trypsinized at 37 °C to release muscle satellite cells. Isolated cells were grown in 100 mm petri dishes (BD Falcon, Franklin Lakes, NJ) in growth medium Advanced MEM supplemented with 10% (vol/vol) FBS, 0.3% (vol/vol) Fungizone, and 0.15% (vol/vol) gentamicin (all obtained from Invitrogen, Paisley, UK) at 37 °C in 5% CO_2_-enriched atmosphere at saturation humidity. Before reaching confluence, cell cultures were purified using MACS CD56 microbeads (Miltenyi Biotec, Bergisch Gladbach, Germany), to separate the myoblasts from other cell types that contaminated the primary cultures. CD56^+^ cells [86] were transferred to new cell culture flasks, and were grown under the same conditions as the primary cultures for two to three more passages, before they were used for experiments.

Differentiation of myoblasts into myotubes was induced with the change of Advanced MEM media supplemented with 10% (vol/vol) FBS to Advanced MEM supplemented with 2% (vol/vol) FBS. Formation of myotubes was observed by bright field microscopy. Well-differentiated cells were observed by bright field microscopy on the 6th day after initiation of differentiation, and electroporation was performed on the 9th day of differentiation. The fusion index of myotubes was 6.08 ± 2.56, the length of myotubes was several hundreds of µm. Electropermeabilization, viability and electrotransfection experiments were carried out on cells plated in Lab-Tek II 4-Chamer slides (Thermo Fisher Scientific, MA; USA).

### Electroporation

In all electropermeabilization, viability and electrotransfection experiments BetaTech pulse generator (BETATECH, Saint-Orens-de-Gameville, France) was used for delivery of high-voltage electric pulses. A pair of parallel wire electrodes with 9.5 mm distance between them (*d*) was used, electrodes were positioned on the bottom edges of the Lab-Tek II 4-Chamber slides to expose all cells to a homogeneous electric field. The electric field (*E*) between the electrodes is due to the geometry homogeneous and can be thus calculated by the formula *E* = *U*/*d*, where *U* denotes the applied voltage and *d* is the distance between the electrodes. In all electroporation experiments we have used a train of 8 consecutive square pulses of 1 Hz repetition frequency. The amplitudes of *E* depended on the pulse length (2 ms or 5 ms). For 8 × 2 ms pulses, the amplitudes of voltages were 100 V, 200 V, 500 V, 600 V, 700 V, 800 V with consecutive applied electric fields *E* = [0.105, 0.21, 0.52, 0.63, 0.73 and 0.84] kV/cm. For 8 × 5 ms pulses, voltages were 100 V, 200 V, 300 V, 400 V, 500 V, 600 V with *E* = [0.105, 0.21, 0.315, 0.42, 0.52 0.63] kV/cm. We used the lower voltages 100 V and 200 V only in preliminary experiments of electropermeabilization to determine the electroporation threshold. For viability and electropermeabilization experiments cells in the control samples (C0) were exposed to the same conditions, but no pulses were applied (*U* = 0 V). Roswell Park Memorial Institute RPMI-1640 (Gennaxon, Germany) medium was used as the electroporation buffer.

### Electropermeabilization

The electropermeabilization threshold (*E*_*c*_), the fraction of permeabilized myotubes and extent of electropermeabilization was obtained using propidium iodide (PI) [5, 64], (Merck/Sigma-Aldrich, Darmstadt, Germany). Briefly, we added PI-a short-term membrane impermeable fluorescent dye in final concentration 0.15 mM in the electroporation buffer (RPMI-1640 medium). Myotubes were electroporated with 8 × 2 ms or 8 × 5 ms pulses with repetition frequency of 1 Hz and increasing voltages. Following pulse delivery myotubes were incubated for 2 min at room temperature to allow PI to enter the permeabilized myotubes. Cells in the control samples (C0) were exposed to the same conditions (added PI), but no pulses were applied (*U* = 0 V). The electroporation buffer with PI was then removed and standard growth media was added. Fluorescent images (Zeiss 200, Axiovert, Germany) were taken at 20 × objective magnification for each sample. Total number of cell nuclei was determined by counting Hoechst positive nuclei. Permeabilization was obtained by counting PI stained nuclei on fluorescent images for each recorded visual field. The fraction of permeabilized cells (*f permeabilization*) was determined from the number of PI positive cell nuclei divided to number of all counted cells nuclei.

We next quantified the extent of myotube electropermeabilization by analyzing the fluorescence intensity of PI *I*_PI_ [a.u.] from the microscopic images with ImageJ software. The extent of permeabilization was calculated as a fraction of maximal increase in the intensity of PI fluorescence:

8 $$f \, PI\_INT_ = \, \frac{{I_{PI} - I_{C0} }}{{I_{max} { + }I_{C0} }} \, , \;$$

where *I*_*C0*_ is the PI fluorescence intensity in the negative control (*U* = 0 V). All experiments were repeated three times. Results from different experiments were pooled together and are presented as mean ± SD.

### Gene silencing of HIF-1α with siRNA electrotransfer

The siRNA against *HIF-1*∝ mRNA (siHIF-1∝) (J-004018-10 from ON-TARGETplus SMARTpool set) was used for gene silencing. The non-targeting scrambled siRNA (siSCR) (D-001810-10-20) (Dharmacon RNAi Technologies, Rockford, IL) was used as control for gene silencing. Before pulse delivery, the growth medium was removed and 200 µl of RPMI-1640 medium containing 10 nM siRNA was added to each well. After 2 min incubation at room temperature, electric pulses were applied. Following the pulse delivery, 25% (v/v) FBS was added. Myotubes were then incubated for 10 min at 37 °C to allow resealing of the plasma membranes, after which 800 µl aMEM with 2% FBS was added. Myotubes were incubated for 24 h at 37 °C in a humidified 5% CO_2_ atmosphere. 24 h after the electroporation, myotubes were treated with 250 μM CoCl_2_ for 4 h to chemically induce the stabilization of HIF-1α protein as described [87]. Whole cell lysates were analysed by western blot (see next section). All experiments were independently repeated three times. Results from different repetitions were pooled together and are presented as mean ± SD.

### Western blot analysis

After the treatment with CoCl_2_, myotubes were washed twice with ice-cold phosphate buffer saline (PBS) and then harvested in Laemmli buffer (62.5 mM Tris-HCl, pH 6.8, 2% [w/v] SDS, 10% [w/v] glycerol, 0.002% [w/v] bromophenol blue, 5% [v/v] β-mercaptoethanol). Total protein concentration was assessed using Pierce 660 nm Protein Assay. Protein samples were separated by SDS-PAGE on 4–12% Bis-Tris gels (Bio-Rad, Hercules, CA), and transferred to PVDF membranes (EMD Millipore, Billerica, MA) using the Criterion system (Bio-Rad, Hercules, CA). To assess sample loading and efficiency of transfer, the membranes were stained with 0.1% (w/v) Ponceau S in 5% (v/v) acetic acid (both from (Merck/Sigma–Aldrich, Darmstadt, Germany). The membranes were blocked in 7.5% (w/v) non-fat milk in TBS-T (10 mM Tris, 137 mM NaCl, 0.02% [v/v] Tween-20, pH 7.6) and subsequently probed with HIF-1α primary antibody (rabbit polyclonal antibody NB100-449, diluted 1:500; Novus Biologicals, Littleton, CO), overnight at 4 °C. Following overnight incubation, the membranes were incubated with the appropriate horseradish peroxidase-conjugated secondary antibody (1706515, diluted 1:15000 Bio-Rad, Hercules, CA). Immunoreactive proteins were visualized using enhanced chemiluminescence (Thermo-Scientific Pierce, Rockford, IL). Agfa X-ray films (Agfa, Zagreb, Croatia) were developed using the Curix 60 developing machine (Agfa HealthCare, Greenville, SC). Quantity-One 1-D Analysis Software (Bio-Rad, Hercules, CA) was used for densitometric analysis. Intensities of individual bands were expressed relative to the total intensity of all the bands (percent adjusted volume). The abundance of actin (rabbit polyclonal antibody SC-1616-R, diluted 1:1000; Santa Cruz Biotechnology, Santa Cruz, CA) was determine as a control.

### Assessment of viability

Electroporation for assessment of viability was performed as described for electropermeabilization (2.3). Immediately after electroporation 25% FBS was added [3, 59]. Following 10 min incubation at 37 °C 800 µl Advanced MEM media with 2% FBS was added. Myotubes were allowed to grow for 24 h at 37 °C in a humidified 5% CO_2_ atmosphere. Myotubes in the control sample (C0) were treated with the same protocol, but without pulse application (*E* = 0 kV/cm). Viability of myotube nuclei was determined by fluorescent microscopy 24h after the electroporation. All myotube nuclei were stained with 2 μg/ml Hoechst 33342 (Life Technologies, Beverly, MA) for 15 min to obtain total nuclei number and with 0.15 mM PI for 5 min to stain nuclei in dead cells, as described previously [88]. At least 20 images at 10 × objective magnification were recorded for each sample using MetaMorph imaging system software (Visitron, Puchheim, Germany). We have determined the number of viable nuclei for each sample by subtracting the number of dead PI-positive nuclei (*PI*) from all Hoechst-positive nuclei (*h*). The fraction of viability (*f Viability*) and percentage of viability in a given sample was determined as the ratio between the number of viable nuclei counted in the treated sample (*N*_*s*_) and the number of viable nuclei in the negative control *C0* (*N*_*0*_):

9 $$\begin{gathered} \hfill \% \,{\text{Viability}}\, = \,\frac{{N_{\text{S}} }}{N_0 } \, \times \,100\, = \,\frac{{h\, - \,{\text{PI}}}}{{h_{{\text{C}}0} \, - \,{\text{PI}}_{{\text{C}}0} }} \, \times \,100 {. } \\ \hfill \, \\ \end{gathered}$$

The results were pooled together and are presented as mean ± SD.

### Gene electrotransfer of pDNA

Plasmid (pEGFP-N1) encoding green fluorescence protein (GFP, excitation 488 nm, emission 507 nm, Clontech Laboratories Inc., Mountain Viw, CA, USA) was used to test electrotransfer of pDNA into primary human myotubes. The plasmid was amplified in DH5α strain of *Escherichia coli* and isolated with HiSpeed Plasmid Maxi Kit (Qiagen, Hilden, Germany). pDNA concentration was spectrophotometrically determined at 260 nm and confirmed by gel electrophoresis. Before pulse delivery, growth medium was removed and 200 µl of electroporation buffer (RPMI-1640) containing 40 µg/ml pEGFP was added to each well. After 2 min incubation at room temperature, electric pulses were delivered. Myotubes were electroporated using a train of 8 × 2 ms pulses with 400 V or 600 V. Immediately after pulse delivery, 25% (v/v) FBS was added. Myotubes were then incubated for 10 min at 37 °C to allow resealing of the plasma membrane, after which 800 μl of aMEM with 2% FBS was added. Following the electrotransfer myotubes were grown for additional 24 h at 37 °C in a humidified 5% CO_2_ atmosphere. Gene electrotransfer was observed by fluorescent microscopy (Zeiss 200, Axiovert, Germany). Bright field and fluorescent images (488/509 nm) of GFP expression were recorded 24 h after electroporation.

### Statistics

One-way analysis of variance (ANOVA), followed by Bonferroni post hoc test was performed to test for differences among groups (control vs treatment). Results are expressed as means ± SD. For analysis of silencing of HIF-1α we have compared level of HIF-1α expression after siRNA delivery compared to delivery of scrRNA (non-targeted scrambled RNA as a control). Statistical analyses were carried out with GraphPad Prism 6 (GraphPad Software, La Jolla, CA). Statistical significance is displayed as follows: ns—not significant (*P* > 0.05); *P ≤ 0.05; **P ≤ 0.01; ***P ≤ 0.001; **** *P* ≤ 0.0001.

### Supplementary Information

**Additional file 1: Figure S1.** Representation in 2D of a spherical cell exposed to the external electric field. The bright shaded part represents the permeabilized surface area Sc, which is exposed to above-threshold transmembrane voltage |U_m_ | > U_c_. **Figure S2.** Western blot images of blots of three independent experiments (N1, N2, N3) for different parameters of electric pulses: trains of 8 x 2 ms and 8 x 5 ms pulses with different voltages. Actin bands are shown as the loading control. SI-siRNA against HIF-1α mRNA, SCR-non-targeting scrambled siRNA.

## Data Availability

All data generated or analyzed during this study are included in this published article.
