# Supplementary material for: Importance of the electrophoresis and pulse energy for siRNA-mediated gene silencing by electroporation in differentiated primary human myotubes
Source: Biomed Eng Online. 2024 May 16;23:47. doi: 10.1186/s12938-024-01239-7 (PMC11097476; doi:10.1186/s12938-024-01239-7)
Supplement: Supplementary file 1 — Additional file 1: Figure S1. Representation in 2D of a spherical cell exposed to the external electric field. The bright shaded part represents the permeabilized surface area Sc, which is exposed to above-threshold transmembrane voltage |Um | > Uc. Figure S2. Western blot images of blots of three independent experiments (N1, N2, N3) for different parameters of electric pulses: trains of 8 x 2 ms and 8 x 5 ms pulses with different voltages. Actin bands are shown as the loading control. SI-siRNA against HIF-1α mRNA, SCR-non-targeting scrambled siRNA. [file 12938_2024_1239_MOESM1_ESM.pdf]

Importance of the electrophoresis and pulse energy for siRNA-mediated gene silencing by electroporation in differentiated primary human myotubes

Mojca Pavlin*, Nives Škorja Milić, Maša Kandušer and Sergej Pirkmajer3*

Supplementary material

Appendix - The induced transmembrane voltage on a spheroidal cell

The induced transmembrane voltage (U_m) of a spheroidal cell placed in an electric field can be calculated either analytically or numerically. We will summarize the main points described in our previous study [58,92]. The most simplified model of a biological cell is a sphere consisting of a cell cytoplasm σ_i surrounded by a very thin, low conducting membrane σ_m, which is placed in a conductive medium σ_e. Analytical solution for the static case for the induced transmembrane voltage U_m is given by Schwan equation[93]:

$$U_m = \frac{3}{2} E_0 R \cos\theta \frac{3\frac{d}{R}\sigma_i \sigma_e}{\sigma_m + 2\sigma_e \quad \sigma_m + \frac{1}{2}\sigma_i \; - \; 1 - \frac{3d}{R} \quad \sigma_e - \sigma_m \quad \sigma_i - \sigma_m}, \qquad \text{(S1)}$$

where d denotes membrane thickness, R cell radius and θ is the angle measured with respect to the electric field direction. For physiological conditions where *the* membrane thickness is much smaller then size of the cells d $<<$ R and $\sigma_m << \sigma_e, \sigma_i$, Schwan equation simplifies into:

$$U_m = 1.5\, E R \cos\theta \; . \qquad \text{(S2)}$$

where R cell radius and θ is the angle measured with respect to the electric field direction. U_m represents the potential drop (induced transmembrane voltage) across the cell membrane. We can define the critical electric field E_c as E where $\theta_c = 0$, therefore:

$$U_c = 1.5\,ER\cos\theta_c\,, \quad E_c = U_c\,/\,1.5R. \tag{S3}$$

The electric field governs the area of the cell membrane S_c, which is exposed to the above-critical transmembrane voltage (brighter shaded region in Fig. S1).

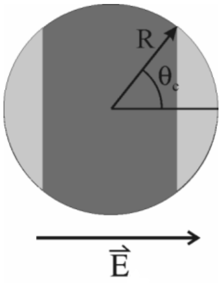

Figure S1: Representation in 2D of a spherical cell exposed to the external electric field. The bright shaded part represents the permeabilized surface area Sc, which is exposed to above-threshold transmembrane voltage |Um | > Uc.

From the above equations we can obtained the permeabilized surface area:

$$S_c = S_0(1 - E_c\,/\,E), \tag{S4}$$

where S_0 is the total surface area of the cell. Clearly, the local electric field E is the critical parameter since it defines the permeabilized area of the membrane $S_c(E)$ and through which molecular transport occurs.

We can extend this to spheroidal shape of the cells (e.g. myotubes). The generalized Schwan's equation for arbitrary oriented ellipsoid can be written:

$$U_m = \sum_{i=x,y,z} r\,E_i \frac{1}{1-L_i}, \tag{S5}$$

where L_i are depolarizing factor in the *x*, *y* and *z* direction and r_i is the vector of the point *T*(*x*, *y*, *z*) at the surface of the spheroid. Here we shall limit ourselves only on axially symmetrical prolate spheroid as a model of myotube $R_1 >> R_2 = R_3$. Depolarizing factor for prolate spheroid along the symmetry axis is

$$L_z = \frac{1-e^2}{2e^3}\left[\log\frac{1+e}{1-e} - 2e\right], \quad e = \sqrt{1-(R_2/R_1)^2}\ . \tag{S6}$$

The depolarizing factors in the other two directions can be calculated from:

$$L_x = L_y = \frac{1}{2}\left(1-L_z\right). \tag{S7}$$

If the *z* axis of the coordinate system is parallel to the symmetry axis of the spheroid then the solution for the parallel and perpendicular orientations are:

$$U_{m\parallel} = z\,E\frac{1}{1-L_z}, \quad U_{m\perp} = x\,E\frac{1}{1-L_x}\ . \tag{S8}$$

Without loss of generality we can always choose so that the vector of the electric field lies in the *xz* plane. The induced transmembrane potential U_m on an arbitrary oriented spheroid can be therefore obtained. The full expression is given in [58], which in cartesian coordinate system simplifies in:

$$U_m = z\,E\cos\alpha\frac{1}{1-L_z} + x\,E\sin\alpha\frac{1}{1-L_x}\ , \tag{S9}$$

where the angle α defines the angle between the electric field direction and the symmetry axis of the spheroidal cell. From the solution for the induced potential in parallel and perpendicular orientation, one can calculate U_m on an arbitrarily oriented spheroid by means of linear

combination of the two solutions. The elongated myotubes can be approximated as prolate ellipsoid with long radius $R_1 \cong 400$ µm and short diameter **20** µm**,** from which we obtain the polarization factors $L_z \cong 0$ and $L_x = L_y \cong 0.5$. Therefore, the maximal induced transmembrane potential is at the poles of the myotubes oriented parallel with E ($\alpha = 0°$) will be:

$$U_{max} = R_1 \times E, \tag{S10}$$

and if we set that $U_{max} = U_c$ then we obtain that :

$$E_c = U_c \;/\; R_1 . \tag{S11}$$

Consequently the longer myotubes oriented parallel to E will be first electroporated. In case that U_c of myotubes would be similar to myoblasts then E_c in myoblast would be lower for the factor of R_1mytoubes/R_1myoblats if all other conditions of the electroporation protocol are the same.

For elongated myotubes the permeabilized surface area S_c increases with the applied voltage/electric field similarly as in Eq. S4 but the function is more complex, details are given in [58]:

$$S_c = R_2 \int_{\tau_1}^{\tau_2} \int_{\varphi_1}^{\varphi_2} \sqrt{R_1^2 \;\; 1 - \tau^2 \;\; + R_2^2 \tau^2}\, d\varphi d\tau . \tag{S12}$$

where φ_1, φ_2, τ_1 and τ_2 are borders of integration defined with the condition that $U = U_c$.

Western blot images (below) of blots of three independent experiments (N1,N2,N3):

N1

8×2 ms　　8×5 ms

500V　600V　700V　800V　300V　400V　500V　600V

1 2 3 4 5 6 7 8 9 10 11 12 13 14 15 16

HIF1a

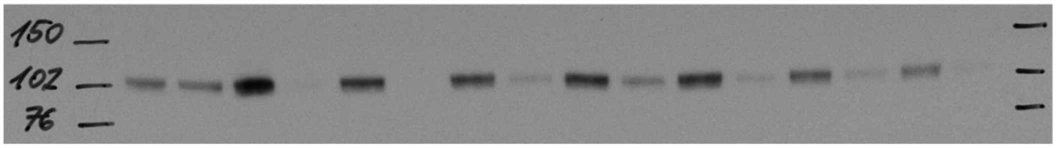

Aktin

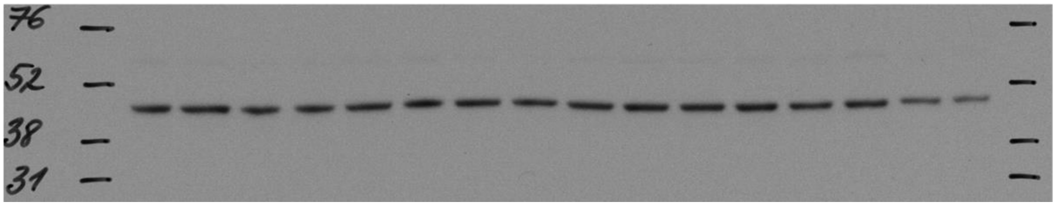

N2

8×2 ms　　8×5 ms

500V　600V　700V　800V　300V　400V　500V　600V

m SCR SI SCR SI SCR SI SCR SI SCR SI SCR SI SCR SI SCR SI m

1 2 3 4 5 6 7 8 9 10 11 12 13 14 15 16

HIF-1α

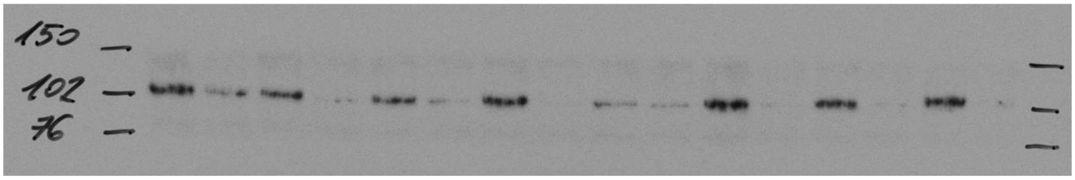

Aktin

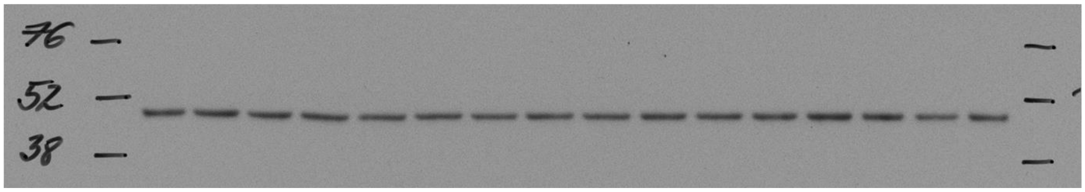

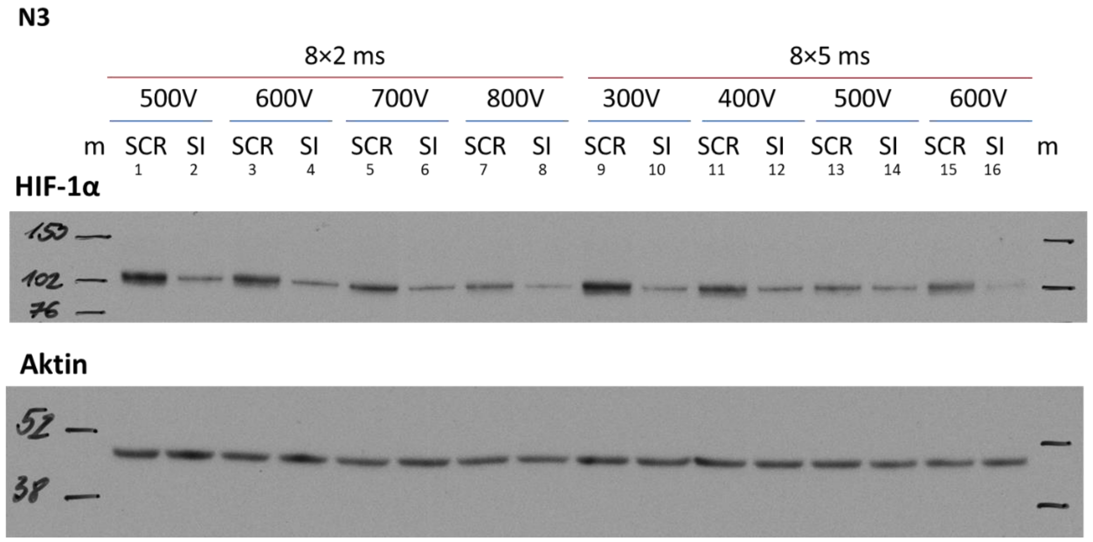

Figure S2: Western blot images of blots of three independent experiments (N1, N2, N3) for different parameters of electric pulses: trains of 8 x 2 ms and 8 x 5 ms pulses with different voltages. Actin bands are shown as the loading control. SI – siRNA against HIF-1α mRNA, SCR - non-targeting scrambled siRNA.
